# Biological motion distorts size perception

**DOI:** 10.1038/srep42576

**Published:** 2017-02-16

**Authors:** Peter Veto, Wolfgang Einhäuser, Nikolaus F. Troje

**Affiliations:** 1 Philipps-University Marburg, Department of Physics, Marburg, D-35043, Germany; 2 Chemnitz University of Technology, Institute of Physics, Chemnitz, D-09107, Germany; 3 Queen’s University, Department of Psychology, Kingston, ON K7L 3N6, Canada

## Abstract

Visual illusions explore the limits of sensory processing and provide an ideal testbed to study perception. Size illusions – stimuli whose size is consistently misperceived – do not only result from sensory cues, but can also be induced by cognitive factors, such as social status. Here we investigate, whether the ecological relevance of biological motion can also distort perceived size. We asked observers to judge the size of point-light walkers (PLWs), configurations of dots whose movements induce the perception of human movement, and visually matched control stimuli (inverted PLWs). We find that upright PLWs are consistently judged as larger than inverted PLWs, while static point-light figures do not elicit the same effect. We also show the phenomenon using an indirect paradigm: observers judged the relative size of a disc that followed an inverted PLW larger than a disc following an upright PLW. We interpret this as a contrast effect: The upright PLW is perceived larger and thus the subsequent disc is judged smaller. Together, these results demonstrate that ecologically relevant biological-motion stimuli are perceived larger than visually matched control stimuli. Our findings present a novel case of illusory size perception, where ecological importance leads to a distorted perception of size.

Systematic distortions in the perception of size can be observed in a wide variety of visual scenarios. Two mechanisms underlie most of the classic examples. One of them is size constancy, where an object that appears farther from the viewer seems to be larger as opposed to a nearer object, even though they create an equally large retinal image in the viewer. The other mechanism is size contrast, where the apparent size of an object changes inversely with the size of other, related, objects. This can take place simultaneously (e.g., a circle among circles in the Ebbinghaus/Titchener illusion), or with a temporal delay (size adaptation aftereffect^1^). Illusions exploiting these mechanisms affect not only the “conscious” percept as reported by the viewer, but also the size of afterimages^2^ or objective measures, such as reaction times^3^,^4^.

While the aforementioned size illusions are perceptual in nature, a different class of size illusions pertains to social constructs that can also lead to a change in perceived size of a person or an inanimate object. A general association between positive subjective value and larger size exists^5^, and this reciprocal connection has been observed in different areas of life. The most palpable example for such a relation is between social leadership and physical size^6^, where it is conspicuous that mechanisms described by evolutionary psychology still play a role in today’s society^7^. Tall men are more likely to take managerial positions than short men^8^, while people with more social power perceive other humans^9^ and objects^10^ as smaller, as well as they are perceived as taller by others^11^ and by themselves^10^. A size-status connection also prevails in the case of consumer products^12^. Aside from power, motivation and action goals^13^ and aesthetic preference judgments^14^ are likewise related to the size of non-animate objects. Altogether, these findings suggest that there is a general, positive, association between the importance or value of an object to the viewer and its perceived size.

Animate motion patterns are rapidly perceivable^15^,^16^,^17^, visually salient^18^ and carry numerous types of information that are readily retrievable by human observers^19^,^20^,^21^. The perception of biological motion is arguably of high ecological importance, making preferential processing by the visual system for such stimuli likely, even though direct evidence is scarce. The most commonly used tool to explore this question is point-light figures. They eliminate all visual information obtainable from the surface of the body by only showing the movements of a few important articulations depicted as dots. With the help of point-light stimuli, biological motion has been shown to yield to several perceptual benefits as compared to similar non-biological motion. For example, coherent and upright point-light walkers (PLWs) are processed incidentally in a flanker paradigm, as opposed to static, scrambled^22^ or inverted^23^ walkers. Upright, scrambled biological motion stimuli lead to faster hits in a search task than similar, but inverted figures^24^, which means that local cues of biological motion act on a preattentive level of visual processing. Upright human or terrestrial animal PLWs induce reflexive attentional orienting in a central cueing paradigm, while inverted or static figures do not^25^, showing that incidental effects are not specific to stimuli presenting configural information that is typical of humans. Indeed, local motion cues, in particular those of the feet, play a crucial role in a “life detector” system: a general filter in human vision, tuned to help us detect terrestrial animals^26^,^27^,^28^.

Biological-motion stimuli, therefore, seem to be of special importance in visual processing. Also, important objects tend to look larger to the viewer. We thus hypothesize that stimuli carrying ecologically valid biological motion cues appear larger to observers, as compared to similar motion stimuli lacking ecological validity. We tested this hypothesis using human PLWs in three experiments, where we compared coherent, upright, PLW figures to inverted ones. In the inverted displays, both local and global biological motion cues lack ecological validity, while all other aspects of the stimulus remain equal to those in the upright figures. Hence, seeing a difference in perceived size between the two conditions can only be due to the effect of the ecological importance of biological motion.

## Experiment I

**Figure 1:**
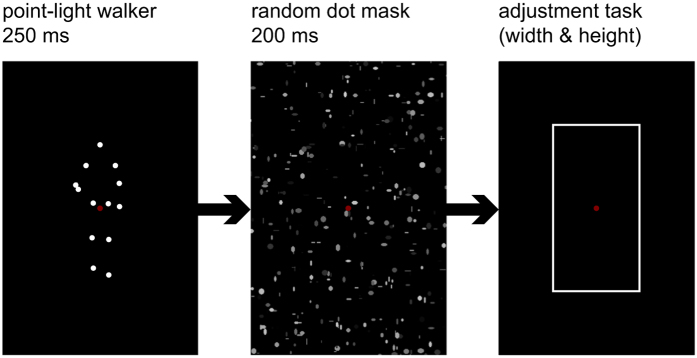
Paradigm–Experiment I. Sequence of a single trial (here with upright PLW).

Perceived sizes of upright and inverted PLWs (see Fig. 1) were compared directly in an adjustment task.

### Methods

#### Participants

Sixteen students from the Queen’s University participant pool (one male, fifteen females, mean age = 20.1, *SD* = 1.8) participated in the study. Experimental protocols of all experiments conformed to the World Medical Association Declaration of Helsinki and were approved by the board “Ethikkommission FB04, Philipps-University Marburg” and by the Human Ethics committee at Queen’s. All participants had normal or corrected-to-normal vision, provided written informed consent and received monetary compensation. One participant dropped out after reporting problems with larger stimulus sizes.

#### Stimuli

Upright and inverted PLWs were depicted from a frontal view, based on the action “Walk” from a stimulus set of human actions created by Vanrie and Verfaillie^29^, based on the actions of a male actor. The figure consisted of 13 dots, showing the positions of the head and the main articulations of the limbs (Fig. 1). Walker size was varied in 10 steps between 2.44° × 0.88° and 7.86° × 2.70° (mean: 5.24° × 1.77°) at a viewing distance of 75 cm. Each PLW presentation started at a random frame of the stride, resulting in slightly varying sizes for each trial. All displays were gray on black background, with a red fixation point continuously shown in the center of the screen. Stimuli were presented on a 17″ CRT screen with Matlab and the Psychophysics Toolbox^30^,^31^.

#### Procedure

For each trial, participants were asked to maintain fixation on the fixation point and viewed a centrally displayed PLW for 250 ms, followed by a dynamic random dot mask, lasting for 200 ms. After the mask, participants had to move the mouse in order to adjust a rectangle to frame the area occupied by the previously seen walker as tightly as possible (Fig. 1). The mouse position was connected to a corner of the rectangle, starting randomly either from the fixation point, or from well outside of the stimulus’ area. The rectangle stayed centrally symmetrical at all times. That way, the width and height of the walker were set independently, albeit in a single response. Participants confirmed their responses by a mouse click, after which the next trial started following a random intertrial interval between 500 and 800 ms. Each participant completed 400 trials.

#### Analysis

For each trial, the percentage of overestimation (area of the response rectangle divided by the area of the smallest frame containing all dots at any time) was calculated. Outlier responses (cutoff = 2.5 SD) were removed for each block (2.2% of all trials). A one sample t-test was carried out to determine whether the difference between responses to upright and inverted walkers (Distortion Effect = Overestimation_Upright Walker_ − Overestimation_Inverted Walker_) was significantly different from zero.

### Results

The size distortion effect was significantly different from zero (expressed in percentage of walker area: mean = 9.07, *SD* = 5.73; *t*(14) = 6.12, *p* < 0.001). This confirms our hypothesis that upright walkers are perceived to be larger than inverted walkers.

## Experiment II

To control whether the observed size-distortion effect is specific to biological motion (rather than an upright/inverted difference per se), we conducted a second experiment similar to Experiment I, with the additional condition of static point-light figures. If the effect is caused by configural information alone instead of biological motion, static figures should elicit the same pattern of results as dynamic PLWs.

### Methods

#### Participants

Twenty-four students from the Chemnitz University of Technology (five males, nineteen females, mean age = 21.9, *SD* = 3.2) participated in the study.

#### Stimuli

Stimuli were presented on a 23.6″ screen (VPixx Technologies Inc., Saint-Bruno, QC Canada), with all other details of the stimulus kept equal to those in Experiment I. In each trial of the additional static condition, a randomly selected frame of the PLW was presented for the same duration of time (250 ms) as the moving PLW in the dynamic condition.

#### Procedure

Each participant completed a total of 640 trials split over four blocks. Two blocks contained dynamic PLWs while the other two contained static point-light figures. Static and dynamic trials were otherwise identical. The order of the four blocks was counterbalanced across observers.

### Results

Dynamic blocks showed a replication of results from Experiment I, with a size distortion effect significantly different from zero (in percentage of walker area: mean = 7.34, *SD* = 15.19; *t*(23) = 2.37, *p* = 0.03). Static blocks on the other hand did not show a significant size-distortion effect (mean = 4.32, *SD* = 17.60; *t*(23) = 1.20, *p* = 0.24).

## Experiment III

Perceived sizes of upright and inverted PLWs were compared indirectly, with a size judgment task on targets presented subsequently to PLWs. As upright PLWs are perceived as larger, we expect that contrast effects will lead to a subsequent target to appear as smaller. Since participants react to simple disc targets and they are instructed to ignore the preceding figures, this experiment further ensures that our previous findings are caused by a perceptual distortion of size and not by any unexplored bias related to PLWs.

### Methods

#### Participants

Sixteen students (five males, eleven females, mean age = 22.1, *SD* = 1.9) participated in the study. Eight (1–8) were measured at Philipps-University Marburg and eight (9–16) at Queen’s University, and recruited through the respective participant pools. All participants had normal or corrected-to-normal vision, provided written informed consent and received monetary compensation.

#### Stimuli

Generation and presentation of stimuli were as described for Experiment I. PLWs (both walkers, in all conditions: 5.4° × 1.9°) and target discs (diameters depending on condition: 0.76° & 0.76°; 0.72° & 0.80°; 0.68° & 0.84°) were presented centered 3.46° above and below fixation. All displays were gray on black background, and a fixation point was continuously shown in the center of the screen.

#### Procedure

**Figure 2:**
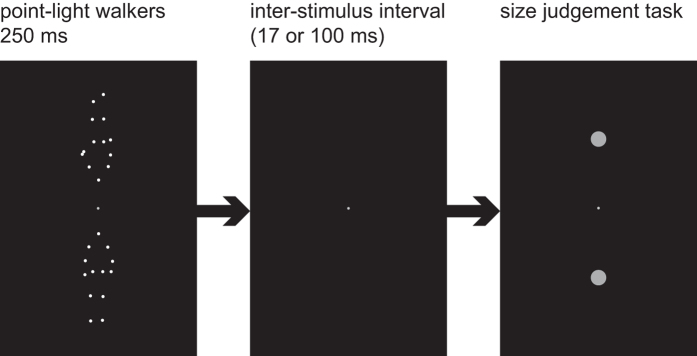
Paradigm–Experiment III. Sequence of a single trial (here with upright PLW in the lower position and inverted PLW in the upper position).

For each trial, participants were asked to maintain fixation on the fixation point while viewing two PLWs (one upright and the other inverted) for 250 ms. Participants were instructed to ignore these displays. Following a blank inter-stimulus interval (ISI) of 17 or 100 ms, two target discs appeared for 100 ms at the locations of the previously seen walkers (Fig. 2). Targets were either identical or differed in size (10.5% or 21% larger or smaller than the average size of 0.76°). Participants gave a non-speeded forced choice response by pressing one of two buttons, indicating which of the targets was larger than the other. After response, the next trial started following a random intertrial interval between 500 and 800 ms.

Participants 1–8 also completed trials for a temporal judgment task in separate blocks, which are not reported here. For participants 1–4, no trials with identical targets were presented. For participants 5–8, eye tracking was used to validate that observers maintained fixation throughout stimulus presentation. Participants 1–4 each performed 400 trials, participants 5–8 each performed 480 trials and participants 9–16 each performed 1000 trials in total.

#### Analysis

**Figure 3:**
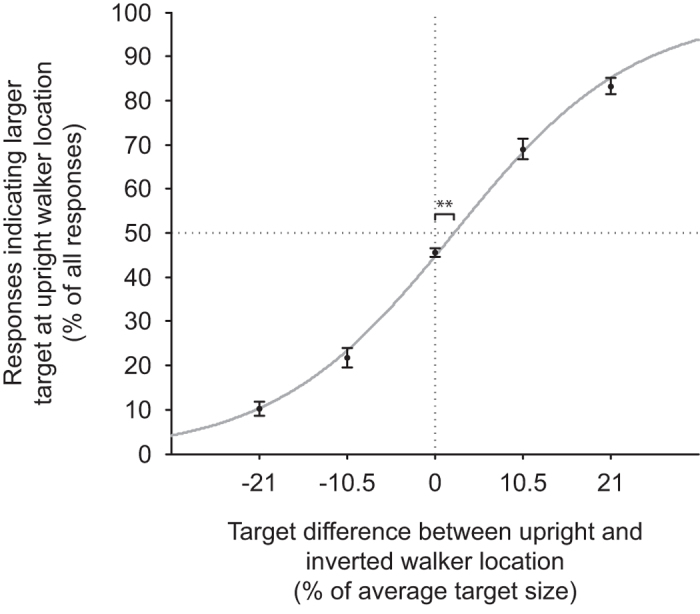
Results–Experiment III. Percent of responses indicating that the target preceded by an upright walker was larger plotted against the difference between target (disc) sizes. Means per condition with fitted psychometric function. Error bars show s.e.m. Asterisks indicate significant difference of PSE at *p* < 0.01. Data for both ISIs (17 ms, 100 ms) were aggregated for analysis. The functional form of the psychometric function is given by f(x; a, l) = a/(a + exp(−l*x)), and thus the PSE by x = −ln[a]/l with fit parameters a and l. Note that the psychometric function for illustration is a fit to the average data, while for statistical analysis each individual was fitted with a separate psychometric function and analysis was based on the distribution of the individual PSEs.

For each participant, the point of subjective equality (PSE) between targets preceded by upright and inverted walkers was calculated. To do so, a psychometric function was fitted to the data of each individual (fraction of responses “larger” at upright PLW location vs. size difference of discs), and the PSE determined analytically from its two fit parameters (cf. Fig. 3). A one-sample t-test was then used to determine whether PSEs were significantly different from zero.

### Results

PSEs were shifted towards larger targets at the upright PLW’s location (mean = 2.60, *SD* = 2.96, in percentage of target size). This shift was different from zero (*t*(15) = 3.51, *p* = 0.003). There was no difference between trials with long and short ISIs (mean_Short ISI_ = 2.63, *SD*_Short ISI_ = 3.26; mean_Long ISI_ = 2.68, *SD*_Long ISI_ = 3.44; *t*(15) = 0.06, *p* = 0.95). This is in line with our hypothesis and shows that targets preceded by an upright walker are perceived as smaller than targets preceded by an inverted walker (Fig. 3).

## Discussion

The findings presented here show that stimuli with ecologically valid biological motion cues appear larger than similar motion stimuli without ecological validity. Experiment I demonstrates this phenomenon. Experiment II replicates the findings and shows that static point-light displays do not lead to a similar distortion in perceived size. Experiment III shows that the effect can also be measured indirectly, as it extends through a contrast mechanism to subsequently presented, neutral, stimuli.

Prior studies have demonstrated that discrimination of biological motion stimuli takes place at an early stage of visual processing^16^ and induces reflexive attentional orienting^25^. This suggests that biological motion stimuli bear high importance, which is further supported by experiments demonstrating that humans^32^ and other animals^33^ have an innate sensitivity to visual invariants characteristic to biological motion. Our findings lead to similar conclusions, as already a brief presentation (250 ms) of biological motion results in a positive distortion of perceived size, which is linked to subjectively important stimuli^5^,^6^,^7^,^8^,^9^,^10^,^11^,^12^,^13^,^14^.

Although a contrast effect seems the most likely mechanism transferring the distortion in perceived size from PLWs to the disc targets used in Experiment III, alternative causes are also possible. For example, spatial attention might be deployed asymmetrically between upright and inverted walkers, causing an inhibition of return^34^ on responses to subsequent target discs. This, however, would not explain the results found in Experiment I & II, where only one, central target is presented at a time.

While PLWs are useful in eliminating surface information from the body, they thus also take biological motion cues out of their natural context. We cannot exclude that from the dots of a point-light figure the perceptual system might “fill in” the rest of the body. If that happens more likely for upright than for inverted figures, a larger percept would be formed for the former. However, Experiment II offers some hint that this may not be the case in our experiments, as the human figure is also clearly recognizable from the frontal view of a static point-light display (cf. Fig. 1).

We cannot exclude that sex differences might also play some role in the results, considering that our participant population was dominantly females and it is conceivable that women are more responsive to biological motion and its social implications^35^. Studies on the link between social power and size^6^,^7^,^8^,^9^,^10^,^11^ suggest that the sex as well as the displayed power of the stimulus figure can likewise affect the outcome. Studying sex differences of the reported effects might therefore be an interesting extension in further research.

As it has been shown with other stimuli already, importance to the viewer makes objects look larger. Our data show that biological motion is no exception. It clearly demonstrates a so far unknown example of distorted size perception. Unlike previous examples, this phenomenon is neither a low-level effect^1^,^2^,^3^,^4^ nor based on social constructs^5^,^7^,9,^10^. Instead, our data suggest that the ecological relevance of a biological motion stimulus makes it incidentally appear larger.

## Additional Information

**How to cite this article**: Veto, P. *et al*. Biological motion distorts size perception. *Sci. Rep.* **7**, 42576; doi: 10.1038/srep42576 (2017).

**Publisher's note:** Springer Nature remains neutral with regard to jurisdictional claims in published maps and institutional affiliations.
